# AKT is indispensable for coordinating Par-4/JNK cross talk in p21 downmodulation during ER stress

**DOI:** 10.1038/oncsis.2017.41

**Published:** 2017-05-22

**Authors:** R U Rasool, D Nayak, S Chakraborty, M M Faheem, B Rah, P Mahajan, V Gopinath, A Katoch, Z Iqra, S K Yousuf, D Mukherjee, L D Kumar, A Nargotra, A Goswami

**Affiliations:** 1 Academy of Scientific and Innovative Research (AcSIR), CSIR-Indian Institute of Integrative Medicine, Jammu, India; 2 Cancer Pharmacology Division, CSIR-Indian Institute of Integrative Medicine, Jammu, India; 3 Department of Biochemistry and Molecular Biology, University of Nebraska Medical Center (UNMC), Omaha, NE, USA; 4 Discovery Informatics Division, CSIR-Indian Institute of Integrative Medicine, Jammu, India; 5 Cancer Biology Division, CSIR-Centre for Cellular and Molecular Biology, Hyderabad, India; 6 Natural Product Chemistry Division, CSIR-Indian Institute of Integrative Medicine, Jammu, India

## Abstract

The double-edged role of p21 to command survival and apoptosis is emerging. The current investigation highlights ER stress-mediated JNK activation that plausibly triggers cell death by attenuating endogenous p21 level. Here, we demonstrated that ER stress activator 3-AWA diminishes the p21 levels in cancer cells by averting the senescent phenotype to commence G2/M arrest. In essence, the deceleration in p21 level occurs through ER stress/JNK/Caspase-3 axis via activation/induction of proapoptotic Par-4 and inhibition of AKT. The molecular dynamics studies identified important interactions, which may be responsible for the AKT inhibition and efficacy of 3-AWA towards AKT binding pocket. Interestingly, the p21 deceleration was rescued by incubating the cells with 3-AWA in the presence of an ER stress inhibitor, Salubrinal. Furthermore, we demonstrated that p21 expression decreases solitarily in Par-4^+/+^ MEFs; albeit, ER stress-induced JNK activation was observed in both Par-4^+/+^ and Par-4^−/−^ MEFs. Par-4 knockdown or overexpression studies established that ectopic Par-4 along with ER stress are not sufficient to downregulate p21 in PC-3 cells but are adequate for DU-145 cells and that the ER stress inflicted activation of JNK, inhibition of AKT and Par-4 induction are all crucial to p21 downmodulation by 3-AWA. By using isogenic cell lines, such as HCT-116 p53^+/+^ and HCT-116 p53^−/−^, we found that deceleration in p21 expression due to ER stress is p53 independent. Moreover, in orthotopic carcinogen-induced rat colorectal carcinoma model, we found that 3-AWA inhibits colorectal tumor growth and formation of colorectal polyps at a tolerable dose, similar to the first-line drug for colorectal cancer-5-fluorouracil.

## Introduction

The diverse biological functions of p21 are well known for its controversial role in predicting the prognosis of cancer patients. p21 levels are highly amplified in various cancers including prostate, cervical, colon, breast and squamous cell carcinomas. p21 activation directly correlates with tumor grade and invasiveness, although the mechanisms remain vague. The oncogenic function of cytoplasmic p21 is exerted through its non-traditional targets in the cytoplasm.^1^ Mechanisms attributed to the pro-survival role of p21 involves G2/M checkpoint regulation, CDKs inhibition, interaction with procaspases and amplifying the HER2 pathway and EMT.^2^ Therefore, the multifaceted network regulating p21 function and its biological activities warrants caution in context to its contribution in apoptosis, genomic stability and senescence. Together, it may not only provide new insights in cancer development but also profoundly impinge on the efficacy of various anticancer drugs that induce p21.

The classical tumor-suppressor role of p21 has been recently complicated by its oncogenic activities.^3^ Cell cycle arrest is an established role of p21 although many studies have reported otherwise. A plethora of reports suggest that p21 protects the cells from stress or p53-dependent and p53-independent apoptosis, although the mechanisms are poorly understood.^4, 5, 6, 7^ Delayed treatment of cancer cells with genotoxic agents causes caspase-3-mediated p21 cleavage and subsequent inactivation, thereby triggering apoptosis.^8^ Moreover, p21 shields the cancer cells from death induced by DNA-damaging agents, and altered p21 expression increases sensitivity to treatment *in vivo*.^9^ Similarly, Martinez *et al.*^7^ reported that low doses of doxorubicin induced p21 expression in human CaP cells, while high doses abrogated p21 expression. Interestingly, p53 expression remained impassive in both stipulations.^6, 7^ Further, the potential significance of p21 in cancer biology has been underscored in human melanoma cells and chronic myeloid leukemia wherein, the comparison between wild-type cells and p21 deleted cells underscored the dual nature of p21 owing to its ability to hamper cell proliferation and drug-induced apoptosis.^10, 11^ Compelling recent evidences have suggested the involvement of AKT in ER stress-induced apoptosis. Hu and colleagues demonstrated sarcoplasmic reticulum calcium pump (SERCA) inhibitor, Thapsigargin or tunicamycin, to transiently activate AKT during ER stress, in MCF-7 cells. The importance of AKT activation as a pro-survival response during ER stress was concreted when MCF-7 cells with suppressed AKT activity were sensitized to ER stress-induced apoptosis.^12^

We have recently demonstrated a prospective therapeutic implication in prostate cancer cells through treatment of these cells by natural product derivative 3-AWA. The presence of α-β-unsaturated carbonyl moiety in natural products bestow them with enhanced chemo-preventive properties, thus rendering a higher degree of specificity to overcome drug resistance.^13, 14^ This withaferin derivative triggered apoptosis by disrupting Bcl2-Beclin 1 interaction through ER stress-mediated Par-4 induction in prostate cancer cells.^15^ 3-Azido withaferin A (3-AWA), a novel derivative of withaferin A, is a strong inducer of Par-4,^16^ and several lines of evidence incriminate the proapoptotic and anti-invasive role of Par-4 in cancer.^17^ In addition, Par-4 suppression in cancer cells has also been implicated in resistance to chemotherapy.^18^ In the current study, we have demonstrated that 3-AWA downregulated p21 via ER stress/JNK/caspase-3 axis. This study, for the first time emphasizes the AKT-dependent cross talk between Par-4 and ER stress in mediating p53-independent p21 downmodulation.

## Results

### ER stress induced by 3-AWA is associated with JNK-dependent p21^Cip1/WAF1^ (p21) downregulation

**Figure 1:**
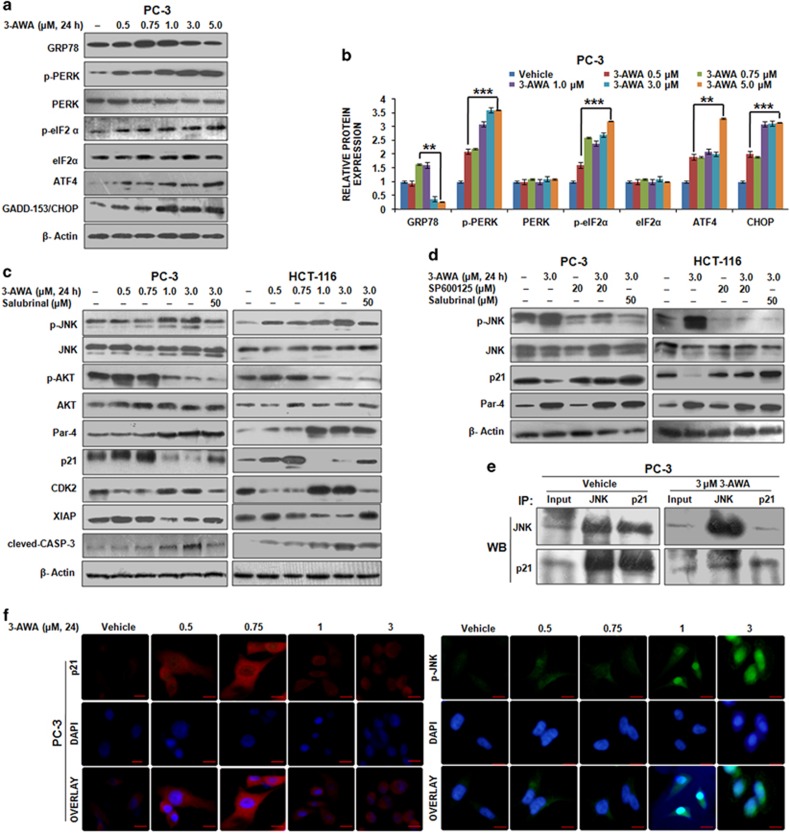
JNK activation and p21^Cip1/WAF1^ (p21) downregulation by 3-AWA. (**a**, **b**) PC-3 cells were exposed to given concentrations of 3-AWA for 24 h. Lysates were prepared and immunoprobed for the indicated proteins through western blotting. The densitometry analysis of western blot results was based on ImageJ software version 1.44p (NIH, USA). (**c**) PC-3 and HCT-116 p53^+/+^ cells were treated with 3-AWA and/or salubrinal for the indicated time point. The protein-specific antibodies were used to analyze the expression of the given proteins through immunoblotting. (**d**) PC-3 and HCT-116 p53^+/+^ cells were treated with different concentrations of 3-AWA and/or SP600125 or salubrinal for the set time point and analyzed for the indicated proteins through western blotting. (**e**) Co-immunoprecipitation (co-IP) assay showing disruption of JNK and p21 interaction by 3-AWA. (**f**) Immunocytochemistry detection of the p21 and p-JNK was carried out in PC-3 cells after treatment with indicated concentrations of 3-AWA. β-Actin was used as loading control. For data analysis, one-way analysis of variance (ANOVA) followed by Tukey’s multiple comparison test was used, bar graphs are mean±s.e.m. Data are the means of three independent experiments. ***P⩽0.001; **P⩽0.01.

Recently, we have reported a consistent induction of proapoptotic protein Par-4 by 3-AWA in prolonged ER stress condition resulting in concomitant blocking of autophagy marker, LC3B-I to LC3B-II conversion, thereby triggering apoptosis through suppression of protective autophagy. Therefore, in the present study, we sought to investigate the comprehensive effects of 3-AWA on ER stress sensors. The results showed that 3-AWA treatment augmented GRP78 levels up to certain concentrations and then gradually diminished. Decrease in GRP78 expression pattern was consistent with an increase in the expression of phospho-PERK1, phospho-eIF2α, ATF4 and GADD153/CHOP (Figures 1a and b). These results may be explained by the fact that during mild to medium ER stress (at lower doses of 3-AWA), the initial protective wing of the UPR pathway gets activated resulting in elevation of GRP78. But as ER stress gets severe, PERK1-mediated activation of ATF4 takes place, which in turn augments the expression of GAAD153/CHOP leading to apoptosis. In the recent past, we have also shown that ER stress induced by 3-AWA is oxidative in nature.^15^ Relevantly, our western blot data showed that 3-AWA induced ER stress was associated with the proapoptotic activation of JNK (Supplementary Figures 1A and B). JNK activation as well as CHOP induction during 3-AWA-induced ER stress might work independently to control apoptosis through Bcl2 family proteins during UPR (Supplementary Figure 1C) and converge to boost apoptotic effect. Given the role of p21 in protecting the cells from apoptosis instigated by stress responses,^1, 19^ we examined the effect of JNK activation by 3-AWA on p21 expression. A sharp induction in p21 was achieved, as confirmed by CDK2 expression at 0.5 and 0.75 μm 3-AWA concentrations, followed by a robust decrease (of p21 levels) at 1 to 3 μm 3-AWA. This was concomitant with the activation of JNK, upregulation of Par-4 and dephosphorylation of AKT in PTEN null PC-3 and Ras mutated HCT-116 cells (Figure 1c). Similar results were obtained in DU-145 cell line (data not shown). This effect was not likely to be due to the proteasome-mediated degradation of p21 because exposure of cells to 5 μm MG132 (proteasome inhibitor) plus 3-AWA treatment had negligible effect in the reduction of p21 expression (Supplementary Figure 1D). Interestingly, the decrease in p21 levels was averted when the cells were subjected to 3-AWA in the presence of an ER stress inhibitor, Salubrinal, (although Par-4 expression in this condition was significantly high as compared with vehicle) suggesting UPR activation and Par-4 induction may simultaneously converge into apoptosis; but when ER stress is blocked, Par-4 could still maintain its elevated level. This may imply a positive outcome of Par-4 induction and ER stress responses in p21 downregulation in maintaining cellular homeostasis. Intriguingly, since high concentration of 3-AWA (3 μm) was sufficient to trigger Par-4 induction^15^ (that too might be independently controlled mechanism), it seems that Salubrinal did not exhibit any pivotal role in maintaining Par-4 levels. On the other hand, elevated Par-4 spontaneously blocked cellular AKT activation,^20^ whereas, as the expression of p21 was reduced, Salubrinal could not prevent 3-AWA mediated caspase-3 activation but blocked the decline in XIAP expression. This provides an evidence that higher concentration of 3-AWA could promote apoptosis even in the presence of salubrinal through XIAP independent pathway. Of note, we checked the expression of AKT, JNK, Par-4 and p21 in salubrinal-treated cells, as given in the Supplementary Figure 1E, and no change in the expression/activation of these proteins was found. The p-JNK-dependent change in p21 expression was confirmed when cells subjected to 3-AWA treatment along with a JNK inhibitor SP600125 abolished the decreasing pattern of p21 expression in PC-3 and HCT-116 cells. This result mimicked the same condition where JNK phosphorylation was abrogated in the presence of 3-AWA and salubrinal, leading to p21 upregulation, indicating ER stress and JNK phosphorylation are intricately corroborated in 3-AWA-mediated p21 downregulation (Figure 1d). The catalytic activity of JNK, a stress-activated MAP kinase concerned with transmitting proapoptotic signals, is reported to be inhibited through its direct sequestration by p21.^21^ Therefore, we sought to investigate the effect of 3-AWA on the interaction between these two proteins in a perspective concerning apoptosis resistance incurred by this interaction of p21 with JNK. The co-immunoprecipitation results demonstrated that a direct interaction between JNK and p21 existed in vehicle-exposed cells. Intriguingly, a drastic loss in the interaction between JNK and p21 was observed in 3-AWA treated PC-3 cells (Figure 1e), underscoring the disruption of JNK-p21 interaction in the apoptosis cascade instigated by 3-AWA. Furthermore, the change in the expression pattern of p21 and p-JNK was analyzed and confirmed through Immunocytochemistry; implicating the diminished p21 fluorescence accompanied the prominent nuclear p-JNK accumulation in dose-dependent 3-AWA treatment (Figure 1f). p21 expressional changes due to 3-AWA treatment was also analyzed in cytosolic and nuclear fractions and it was found that p21 levels were elevated both in cytosol and nucleus initially, but decreased at higher concentration of 3-AWA (Supplementary Figures 1F and G). In addition to the evident link of the apoptotic morphology of the cells with the downregulation of p21, contrastingly, a senescent type of morphology, as evident by β-galactosidase staining (Supplementary Figures 1H and I) was also identified during p21 upregulation at lower doses of 3-AWA. As translational halt caused by eIF2α phosphorylation in response to various stresses decides whether global protein synthesis and cell proliferation should continue or not,^22^ we checked the effect of 3-AWA on the expression and activation of eukaryotic translation initiation factor-4E (eIF4E) in HCT-116 cells. Although 3-AWA was initially found to increase the phosphorylation of eIF4E, at higher concentrations, eIF4E dephosphorylation (inhibition of protein synthesis) accompanied by decreased expression of GRP78 and increased expression of p-eIF2α were observed. Interestingly, in the presence of salubrinal, 3-AWA could not inhibit the activity of eIF4E, as evident by its phosphorylation levels (Supplementary Figure 1J), signifying that it is indeed 3-AWA induced UPR, through which eIF2α regulates eIF4E activity (ongoing study in our laboratory). Collectively, these results advocate that the coordinated stimulation of ER stress (by 3-AWA) activates the JNK-mediated apoptotic arm of UPR leading to p21 downmodulation, which is known to perturb cellular homeostasis.

### p21 downregulation by 3-AWA is mediated via ER stress/JNK/caspase-3 axis

**Figure 2:**
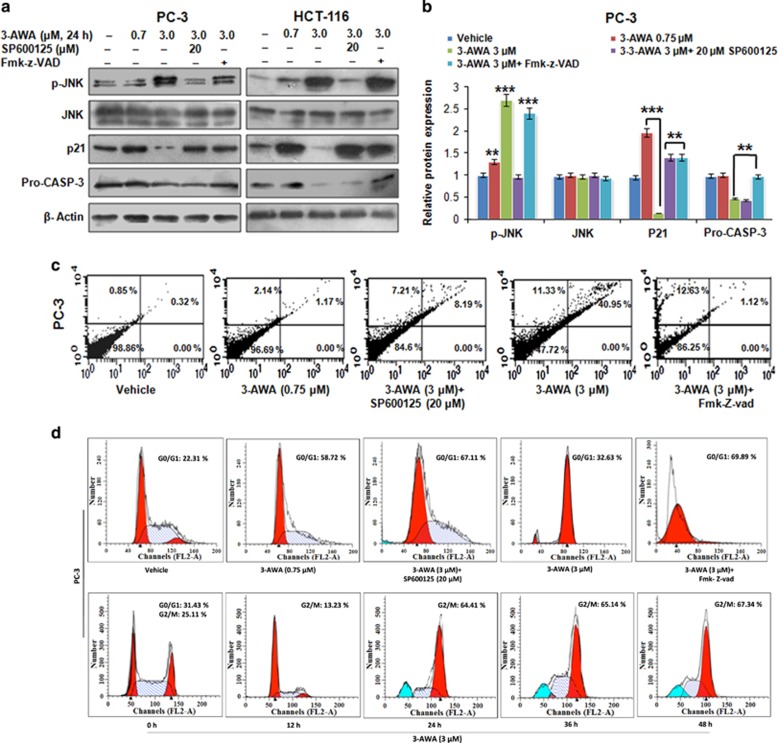
p21 downmodulation by 3-AWA through ER stress/JNK/caspase-3 cascade. (**a**, **b**) Immunoblotting analyses of PC-3 and HCT-116 p53^+/+^ cells showing the expression of the indicated proteins after the cells were treated with 3-AWA and/or SP600125 or Fmk-z-VAD. (**c**) Annexin V-FITC analysis of PC-3 cells, following treatment with 3-AWA and/or SP600125 or Fmk-z-VAD for 24 h, was carried out to check percentage of apoptosis. (**d**) Cell cycle analysis was performed in PC-3 cells treated with 3-AWA alone and/or SP600125 or Fmk-z-VAD for a fixed time point of 24 h (upper panel) or in a time point dependent manner (lower panel). β-Actin was used as loading control in western blot experiments. All experiments were performed in triplicates. Statistical significance was calculated by one-way analysis of variance (ANOVA) followed by Tukey’s multiple comparison test, bar graphs are mean±s.e.m. ***P⩽0.001; **P⩽0.01.

p21 negatively modulates the threshold of apoptosis induced by oxidative stress or growth factor withdrawal.^4, 23^ JNK is activated by the ER stress transducer PERK and induces apoptosis under prolonged ER stress.^24, 25^ This observation in association with the antiapoptotic effect of p21, prompted us to hypothesize that 3-AWA mediated JNK activation might regulate p21 expression to facilitate cell death. Therefore, we investigated the detailed mechanistic pathway how p21 was downregulated during sustained ER stress by potential anticancer agent 3-AWA. Necessarily, the cells were treated with 3-AWA and/or JNK inhibitor SP600125 or caspase inhibitor Fmk-z-VAD and successively analyzed for p21 expression. The 3-AWA arbitrated p21 modulation was completely suppressed by SP600125 as well as by Fmk-z-VAD in all the three cell lines *viz* PC-3, HCT-116 (Figures 2a and b) and DU-145 cells, respectively (Supplementary Figure 2A). These data prompted us an impression that it was indeed ER stress-mediated JNK activation through which caspase-3 cleavage was downmodulating p21 expression. To correlate the JNK-mediated p21 expressional changes (in presence of 3-AWA) with apoptosis, using the similar conditions, we carried out Annexin V-FITC apoptosis assay and the results unveiled that majority of the cells entered the apoptotic quadrant as the p21 expression attenuated (Figure 2c). When investigating the effect of p21 downregulation by 3-AWA on cell cycle progression; the arrest of PC-3 and HCT-116 (data not shown) cells in G1 phase in 3-AWA plus SP600125 and/or Fmk-z-VAD-treated cells was noted. This result could be justified as 3-AWA alone treated cells exhibited a reduced p21 expression and G2/M phase arrest following 24 h treatment (Figure 2d). The time-dependent pattern of p21 expression, following 3-AWA treatment, supported our cell cycle analysis data, wherein the expression of p21 enhanced at 12 h and then drastically decreased after 24 h (Supplementary Figure 2B). Further, the colony-formation assay also confirmed that the inhibition of cell proliferation was through 3-AWA-mediated regulation of ER stress/JNK route (Supplementary Figure 2C). Together, the above studies highlight the importance of a novel ER stress/JNK/caspase-3/p21 cascade, exploited by 3-AWA to abrogate the resistance to apoptosis incurred by p21 in prostate/colon cancer cells to cellular stress.

### Dephosphorylation of AKT by 3-AWA co-ordinates Par-4/JNK cross talk in p21 downmodulation

**Figure 3:**
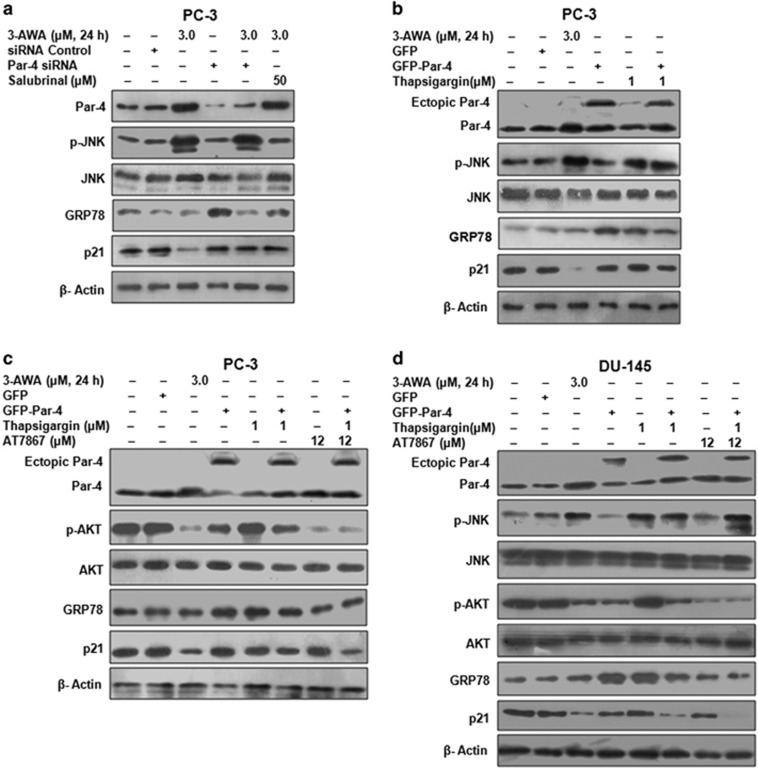
AKT inhibition by 3-AWA co-ordinates Par-4/JNK intrication in p21 downmodulation. (**a**) PC-3 cells were transiently transfected with siRNA control or Par-4 siRNA and then treated with 3-AWA and/or Salubrinal for 24 h. The expression of the indicated proteins was analyzed through western blotting. (**b**) PC-3 cells were transiently transfected with GFP or GFP-Par-4 and then treated with 3-AWA and/or Thapsigargin for the set time point. The lysates were prepared and subjected to western blotting for the expressional analysis of Par-4, p-JNK, JNK and p21. (**c**) Immunoblotting analysis of Par-4, p-AKT, AKT and p21 was carried out in PC-3 cells, transiently transfected with GFP or GFP-Par-4, then treated with 3-AWA and/or Thapsigargin or an AKT inhibitor AT7867. (**d**) Lysates prepared from DU-145 cells, transiently transfected with GFP or GFP-Par-4 following treatment with designated concentrations 3-AWA and/or Thapsigargin or AT7867 for the indicated time point, were subjected to western blotting for the detection of Par-4, p-AKT, AKT, p-JNK, JNK, GRP78 and p21. β-Actin was used as loading control and the data shown are the representative of three independent experiments.

3-AWA is a strong inducer of a proapoptotic tumor-suppressor protein Par-4.^16^ Therefore, inhibition of 3-AWA-mediated p21 downmodulation by neutralizing ER stress (through Salubrinal/Figure 1c) prompted us to dissect any relation between ER stress and Par-4 in regulating 3-AWA-mediated p21 downregulation. We carried out Par-4 overexpression and knockdown studies in presence of Salubrinal. The results implied a sharp decline in JNK activation by 3-AWA in Salubrinal-treated wells, whereas there was negligible effect of Par-4 knockdown on JNK activation by 3-AWA. Interestingly, 3-AWA arbitrated inflection of p21 was inhibited in Par-4 knockdown as well as in Salubrinal-treated cells (Figure 3a). To further explore any intrication between Par-4 and ER stress in mediating p21 downregulation, we performed Par-4 overexpression studies in presence of 3-AWA and/or ER stress inducer Thapsigargin. The results depicted a persistent JNK activation caused by Thapsigargin, but negligible decline in p21 expression was achieved in Thapsigargin treated Par-4 overexpressed cells. GRP78 protein levels were examined to assess the extent of ER stress caused by 3-AWA and that of Thapsigargin (Figure 3b). However, previously Chang *et al.*^26^ have reported that phosphorylation of p21 (T145) by AKT is vital to retain p21 in the cytoplasm and execute its antiapoptotic activities. Furthermore, AKT hyperactivation also sequesters Par-4 to regulate it negatively.^13^ Therefore, we assumed that inhibition of AKT was required to intercede an intrication between Par-4 and ER stress/JNK in p21 regulation by 3-AWA. To validate this, western blot analysis of Par-4 overexpressing cells, incubated with 3-AWA/Thapsigargin and/or AT7867, showed that Thapsigargin and Par-4 alone or in combination and Thapsigargin plus AT7867 were unable to reduce p21 expression markedly in PC-3 cells, but did so efficiently by an assemblage of Par-4, AT7867, and Thapsigargin (Figure 3c). This was further substantiated by exploiting the same conditions in DU-145 cells. In contrast, we found that only ectopic Par-4 along with Thapsigargin had a significant effect on p21 downmodulation and further inhibition of AKT by AT7867 showed an added effect to this p21 wipe off (Figure 3d). Of note, similar to PC-3 cells (data not shown), AT7867 has no effect on phosphorylation of JNK in DU-145 cells. These results implicated that Par-4 overexpression along with ER stress are not sufficient to downregulate p21 in PC-3 cells but are for DU-145 cells due to the differential PTEN status in these two cell lines. Interestingly, these results also inferred the role of AKT dephosphorylation in mediating p21 downregulation by 3-AWA through a connection involving Par-4 and ER stress. This JNK activation and p21 downmodulation with 3-AWA was also carried out in Par-4^+/+^ and Par-4^−/−^ MEFs. It was found that p21 expression decreased solitarily in Par-4^+/+^ MEFs, albeit ER stress-induced JNK activation was observed in both Par-4^+/+^ and Par-4^−/−^ MEFs indicating although JNK activation is a prerequisite but not sufficient to modulate p21 unless the AKT activity is blocked (Supplementary Figures 3A and B). Here, we show that 3-AWA simultaneously exhibited two pivotal roles: first, it caused UPR/ER stress-mediated JNK phosphorylation and second, it abrogated the AKT activation, which are both decisive for antitumor activities. Thus, the above studies underline the ER stress inflicted activation of JNK, inhibition of AKT and Par-4 induction crucial for p21 downmodulation by 3-AWA.

### Deceleration in p21 expression by 3-AWA pursues a p53-independent mechanism

**Figure 4:**
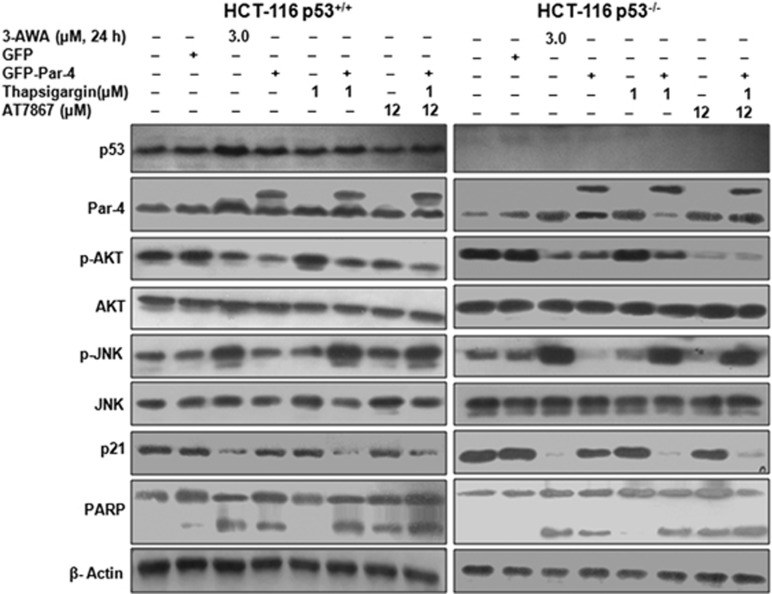
p21 downregulation by 3-AWA occurs in p53-independent manner. HCT-116 p53^+/+^ and HCT-116 p53^−/−^ cells, transiently transfected with GFP or GFP-Par-4 and then treated with indicated concentrations 3-AWA and/or Thapsigargin or AT7867 for the designated time point, were subjected to immunoblotting for the detection of Par-4, p-AKT, AKT, p-JNK, JNK, p53, PARP and p21. Representative data from at least three independent experiments are shown.

The development of fatal human cancers is intricately associated with the loss of p53 or mutations in p53.^27, 28^ The relationship between p53 status, its control on p21 and the sensitivity of cancer cells to a variety of drugs are important prognostics in cancer development and progression.^25^ A handful of evidences suggest that in DNA-damaging response of human fibroblasts, p21 upregulation confers protection against apoptosis and renders them to undergo accelerated senescence.^29^ So far, we have demonstrated how 3-AWA abrogated the resistance to apoptosis incurred by p21 in p53 null PC-3 cells, by sequestering its (p21) cytoplasmic accumulation. To check whether the downstream p21 suppression in this potential ER stress/JNK/caspase-3 axis (due to 3-AWA treatment) is independent of the cellular status of p53, we used isogenic HCT-116 p53^+/+^ and HCT-116 p53^−/−^ colon cancer cells. We observed that similar to DU-145 cells, Thapsigargin and Par-4 combination had a profound effect on p21 expression in both HCT-116 p53^+/+^ and HCT-116 p53^−/−^ cell lines, which was further amplified with the addition of AT7867 (Figure 4). Moreover, a noticeable increase in PARP cleavage was also evident with the decrease in p21 expression by 3-AWA. In addition, caspase-3/7 activation, under the conditions tested in Figure 4, was more profound in the conditions where p21 was downmodulated (Supplementary Figures 4A and B), thus signifying the role of p21 in apoptosis resistance mechanisms. Thus, 3-AWA at its subtoxic doses is capable of inducing p53-independent p21 expression and, hence, premature senescence (data not shown). In contrast, at its sublethal doses, 3-AWA sensitizes cells by switching on apoptosis through p21 downregulation.

### Molecular dynamics simulation of 3-AWA–AKT interaction

**Figure 5:**
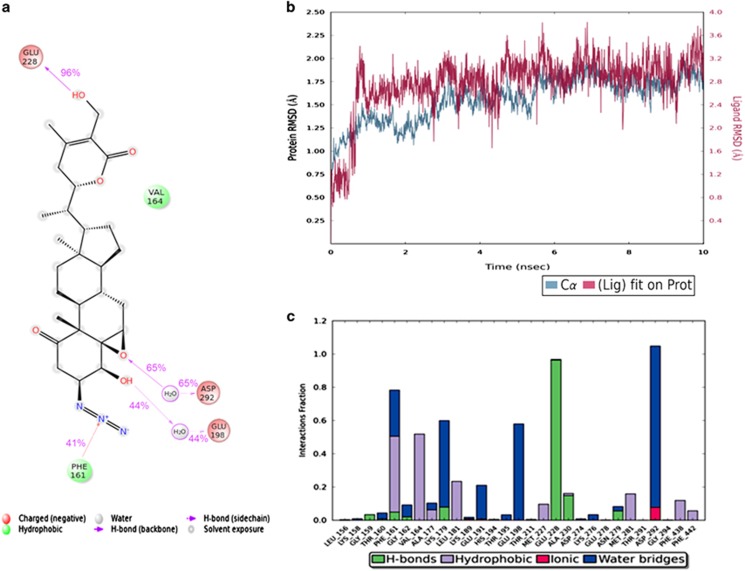
Interglot of MD simulation runs of molecule 3-AWA with AKT. (**a**) Protein–ligand contacts and (**b**) protein–ligand RMSD. (**c**) Detailed interaction of ligand atom with the protein residues occur more than 30.0% of the simulation time is shown.

Given the above data, we sought to identify how 3-AWA interacts with AKT at the molecular level. By the MD simulation run, it was identified that the Asp292 and Glu198 amino acid residues of AKT were interacting with 3-AWA via formation of H-bonding with the water residues. These interactions persisted for 65% and 44%, respectively during the simulation run of 10 ns. Glu228 forms direct H-bonding with OH group for 96% during the simulation, whereas Phe161 provided additional stability to the protein–ligand complex via forming a pi-cation interaction with the charged N atom as shown in Figure 5a. The RMSD plot of protein–ligand complex depicted that both the protein and the ligand were stable during the whole simulation run and form a stable complex (Figure 5b). From the protein–ligand contacts, as shown in Figure 5c, it was identified that an additional ionic interaction of Asp292 occurred with the molecule, which persisted for less than 15% during the simulation which provided an additional stability to the complex. Thus, from molecular dynamics studies, important interactions (3-AWA-AKT) were indentified, which may be responsible for the enzyme inhibitory activity and efficacy of the molecule towards AKT binding pocket.

### 3-AWA inhibits colorectal tumor growth and formation of colorectal polyps

**Figure 6:**
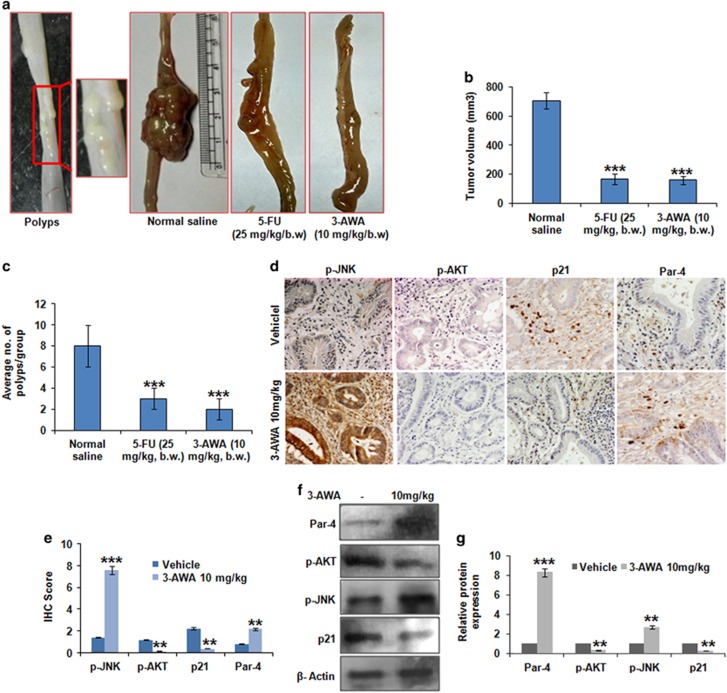
Efficacy of 3-AWA in orthotopic carcinogen-induced rat colorectal carcinoma model. (**a**) Colorectal polyps were induced in male Wistar rats as described in the 'Materials and methods' section; after sufficient tumor growth, the animals received 10 mg/kg b.w. of 3-AWA, the positive control group received 25 mg/kg b.w. of 5-FU and the negative control group animals were given 0.9% normal saline solution in alternate days for 3 weeks. The animals were then killed and tumor growth was measured. (**b**) Bar graphs showing tumor volume in each group of animals (*n*=7 animals per group). (**c**) Bar graphs showing number of polyps in each group of animals. (**d**, **e**) For immunohistochemical detection of the p-AKT, p-JNK, Par-4 and p21, paraffin-embedded sections from the tumors of treated and untreated mice were incubated with respective primary antibodies and were exposed to 3,3′-diaminobenzidine (DAB) substrate, counterstained with hematoxylin and finally dehydrated and mounted for confocal microscopy. The scoring was done by two independent pathologists. (**f**, **g**) Tumors excised from controls and treated animals were homogenized and sonicated in lysis buffer. Lysates were prepared and processed for western blotting as shown. For data analysis, Student's *t*-test and one-way analysis of variance (ANOVA) with SAS 9.2 were used, bar graphs are mean±s.e.m. *In vitro* as well as *in vivo* data are the means of three independent experiments. ***P⩽0.001; **P⩽0.01.

To evaluate the antitumor efficacy of 3-AWA, we used orthotopic carcinogen-induced rat colorectal carcinoma model. We observed sufficient tumor growth in all the six animals of the control group (mean tumor volume: 705 mm^3^) along with colorectal polyps throughout the colorectal mucosa. However, the mean tumor volume reduced significantly in 3-AWA-treated (10 mg/kg of body weight (b.w.) group (158 mm^3^) compared with the vehicle-treated control group. Post treatment, the positive control 5-FU-treated (25 mg/kg b.w.) group contained mean tumor volume of 165 mm^3^. On the other hand, the number of polyps reduced significantly (6 to 10 polyps/rat in the control group versus one to three polyps per rat in treatment group) in the 3-AWA- as well as 5-FU-treated group (Figures 6a–c). The tumors were plaque-shaped or polyploid and localized in the mucosa of the large bowel. The body weight was increasing gradually until the termination of the experiment; no mortality and significant body weight loss was observed in the animals throughout the experimental period (Supplementary Table). After dissection, the colorectal tumor samples were subjected to immunohistochemistry analysis. The results depicted a significant increase in Par-4 and p-JNK expression with the concomitant decrease in p21 and p-AKT expression, as evidenced by the IHC scoring results (Figures 6d and e). Western blot analysis of the tumor samples was carried out and it mimicked our immunohistochemistry results (Figures 6f and g). Thus, the data clearly demonstrate that 3-AWA inhibits colorectal tumor growth and formation of colorectal polyps at a tolerable dose of 10 mg/kg, similar to the first-line drug for colorectal cancer-5-fluorouracil.

## Discussion

Significant advances have been made to elucidate the key players that affect the regulation and cellular localization of p21. In the current study, we demonstrate that pharmacological induction of ER stress by 3-AWA attenuates p21 levels, an effect that coincides with the activation of JNK. We have also provided evidence that Par-4, a major player contributing to cancer cell apoptosis, may be involved in the regulation of the cell cycle regulator p21 during ER stress facilitating the commitment of cells to a proapoptotic program. Our findings are consistent with a mode of action of subtoxic doses of 3-AWA, at which endogenous p21 induces cell cycle arrest and inhibits apoptosis instigating the pro-survival processes during the initial stages of ER stress. However, a higher concentration of 3-AWA promotes p-JNK-dependent abrogation of p21 expression levels leading to Par-4-mediated proapoptotic effects of ER stress.

3-Azido withaferin A (3-AWA), a derivative of the α-β-unsaturated functionality of ring A of withaferin A, has been well documented for its antiproliferative potential and is superior to its parent compound, withaferin A, in stalling cancer progression.^18^ Although, 3-AWA exerts strong antiproliferative activity in various cancer types, until now the detailed ER stress-mediated mechanism of its apoptosis induction function has not been studied. Based on our previous studies, we found that 3-AWA induced ER stress activates JNK, which is associated with Fas upregulation. Whether JNK possesses a pro-survival or a pro-death role is still a debatable issue.^30, 31, 32, 33^ Temporal regulation of JNK may also be a decisive factor of cellular responses; while transient JNK activation causes cell survival, prolonged activation is known to trigger apoptosis.^34^ JNK is activated by the IRE1–TRAF2–ASK1 branch of the UPR.^35^ Here, we have shown that initially, up to a certain concentration of 3-AWA, p21 expression elevated gradually, but as soon as ER stress reached to its UPR level, p21 sharply attenuated along with JNK phosphorylation. Recent report implied that during ER stress the expression of p21 is abolished through CHOP-dependent suppression of its promoter-activity^36^ and CHOP-mediated apoptosis is associated with the suppression of antiapoptotic protein, p21.^37^ Compelling evidences suggest that p21 may be downmodulated by a mechanism, which operates through ER stress/JNK/casp-3 axis, but how this cascade is toggled is not clear.^7^ Here, for the first time, we distinctly prove that phosphorylation status of AKT is a key factor in favoring an association between Par-4 and JNK to modulate p21 expression in ER stress condition. These findings may provide an insight into the probable role of Par-4 in cell cycle regulation.

c-Jun N-terminal kinase (JNK) is considered as a major downstream activator in stress responses. JNK activation in response to diverse forms of stress is noted to impact the cell death machinery by regulating the Bcl2 family proteins.^38^ Inhibitor of the c-Jun N-terminal kinase (JNK), SP600125, has been demonstrated to increase p21 expression and promote p21 phosphorylation via PI3K/AKT pathway, thus preventing p21 binding to PCNA and direct inactivation of caspase-3.^39^ Herein, we found that 3-AWA arbitrated p21 modulation was inhibited by SP600125 as well as by Fmk-z-VAD in the presence of 3-AWA, implying ER stress-mediated JNK activation rendered the suppression of p21 via caspase-3 stimulation (Figure 2a). In addition, a correlation between p21 expressional change due to 3-AWA and apoptotic threshold was confirmed when we noticed a stumpy p21 expression after 24 h concomitant with G2/M phase arrest, implicating the role of 3-AWA-mediated activation of ER stress/JNK/caspase-3/p21 cascade in preventing the resistance towards apoptosis incurred by p21 in proliferating cells.

Agents that selectively restrain AKT activation may not only divest tumors of the pro-survival functions of AKT but are also likely to subvert p21 leading to elevated apoptotic effects. A study where the DNA-damaging agent aminoflavone induced apoptosis of MCF-7 breast cancer cells by simultaneously blocking AKT activity as well as p21 stabilization supports this possibility.^3^ As reported previously, phosphorylation of p21 (T145) by AKT is vital to retain p21 in the cytoplasm and execute its antiapoptotic activities.^26^ AKT hyperactivation also confiscates Par-4 and regulates it negatively and we have shown recently that 3-AWA dephosphorylates AKT and favors threshold induction of Par-4.^18, 40^ But in the current consequence, we found that Par-4 induction and stimulation of ER stress on their own are not sufficient to destabilize p21. We expected that inhibition of AKT was too required to intercede an intrication between Par-4 and ER stress/JNK in p21 regulation by 3-AWA. Indeed, we found that the ER stress inflicted activation of JNK, AKT inhibition, and Par-4 induction are equally crucial to p21 downmodulation by 3-AWA in aggressive cancer cells. However, keeping in view that 3-AWA is a strong Par-4 inducer as well as a negative regulator of antiapoptotic p21, we investigated the physiologically relevant effects of 3-AWA on orthotopic tumor growth and we found that 3-AWA inhibits colorectal tumor growth and formation of colorectal polyps at a tolerable dose of 10 mg/kg, which was similar to the first-line drug for colorectal cancer-5-fluorouracil.

**Figure 7:**
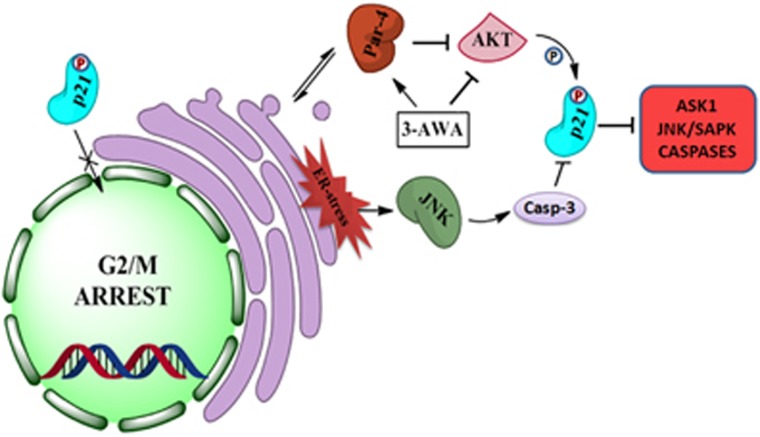
Schematic representation of the regulation of p21 by 3-AWA. 3-AWA induces Par-4 and stimulates ER stress-mediated activation of JNK. The simultaneous inhibition of AKT by 3-AWA favors a cross talk between Par-4 and JNK to downmodulate p21 through AKT/Par-4/JNK/caspase-3 axis.

In conclusion, our findings unveil a novel mechanism of p21 regulation involving AKT/Par-4/JNK axis in the most aggressive type of cancers, as shown in Figure 7. Elucidation of this axis might lead to improvement of existing therapeutic regimens, which are mainly operating through DNA-damaging response or the ones targeting cell cycle regulation.

## Materials and methods

### Cell culture and reagents

The prostate cancer cell lines PC-3 and DU-145 were purchased from the ATCC; Dr Vivek Rangnekar, University of Kentucky, Lexington gifted *Par-4*^*+/+*^ and *Par-4*^−/−^ MEFs; Dr Bert Vogelstein, Johns Hopkins University, Baltimore, MD gifted p53^+/+^ HCT-116 and p53^−/−^ HCT-116 cell lines. These cell lines were cultured in RPMI 1640/DMEM medium (Invitrogen Life Technologies, Carlsberg, CA, USA) supplemented with 10% fetal bovine serum, 70 mg/l penicillin, 100 mg/l streptomycin, 6 mm HEPES and 2 mm l-glutamine (Sigma-Aldrich, St Louis, MO, USA) at 37 °C and 5% CO_2._ The major inhibitors including ER stress inhibitor Salubrinal, JNK inhibitor SP600125 were purchased from Selleck Chemicals, Houston, TX, USA. AKT inhibitor AT7867, caspase inhibitor Fmk-z-VAD and ER stress inducer Thapsigargin were procured from Santa Cruz Biotechnology, Inc., Santa Cruz, CA, USA.

### Western blot and co-immunoprecipitation analysis

Following the treatments, the cell lysates were prepared with TEGN buffer (10 mm Tris-HCl pH 7.5, 1 mm EDTA, 400 mm NaCl, 0.5% NP-40 and 1 mm DTT) supplemented with protease inhibitor cocktail. Western blotting was carried out as described.^41^ The antibodies used—anti-phospho-JNK (#4668S), anti-phospho-AKT (#9271S), anti-phospho-PERK (#3191S), anti-eIF2α (#9722S), anti-phospho-eIF2α (#3597S), anti-Bcl2 (#2872S), cleaved-caspase-3 (#9661S) were purchased from Cell Signalling Technologies, Beverly, MA, USA; pro-caspase-3 (ab44976, Abcam, Cambridge, UK); anti-Par-4 (sc-1807), anti-p21 (sc-817), anti-AKT (sc-8312), anti-PERK (sc-13073), anti- FAS (sc-714), anti-CDK2 (sc-748), anti-XIAP, anti-PARP (sc-7150), anti-GRP78 (sc-13968), anti-JNK (sc-572), p53 (sc-6243), anti-GAAD (sc-575) antibody were from Santa Cruz Biotechnology, Inc. Anti-β-actin (A5316), anti-Rabbit (A6154) and anti-Mouse (A4416) monoclonal antibody were purchased from Sigma-Aldrich. The quantitative analysis of western blot results was based on ImageJ software version 1.44p (NIH, USA). For co-immunoprecipitation, lysates were precleared by adding 20 μl of normal IgG conjugated to 50 μl of protein G-Sepharose beads. Twenty-five microliters of antibody conjugated to 50 μl of protein G-Sepharose beads was used to immunoprecipitate the precleared lysates.

### Plasmids and transfections

Plasmids expressing GFP-Par-4 or GFP were purchased from addgene (Addgene, Cambridge, MA, USA). Transfections were carried out with Lipofectamine 2000 or Neon Transfection system (Invitrogen Life Technologies) as per the manufacturer’s protocol and as described previously.^18^

### siRNA transfection

Par-4 siRNA was obtained from Dharmacon (Lafayette, CO, USA) and briefly, desired number of cells was seeded in appropriate dishes or wells and transfected with human specific Par-4. Oligofectamine (Invitrogen Life Technologies) was used for transfections as per the manufacturer's guidlines.

### Immunocytochemistry

For p-AKT and p21 protein immunostaining, the cells were seeded in the Lab-Tek chamber slides (Thermo Scientific, Boston, MA, USA) at a seeding density of 5 × 10^4^ cells/chamber. Following transfections or treatments with 3-AWA, the cells were incubated for set time points and immunocytochemistry was performed as described previously.^17^ The prepared slides were then analyzed with Zeiss LSM-510 metaconfocal microscope under × 40 magnification (scale used was 100 μm).

### Cell cycle analysis

Cell cycle analysis was done as per described protocol.^42^ After treatment for set time points, the cells were collected and incubated in a hypotonic solution (0.1% sodium citrate, 25 μg/ml propidium iodide, 0.03% Triton X-100 and 40 μg RNase-A) for 30 min at room temperature in the dark. BD Diva software (BD, San Jose, CA, USA) was used for the subsequent FACS analysis.

### Immunohistochemistry

For immunohistochemical detection of the specific proteins, paraffin-embedded sections from the tumors of treated and untreated mice were prepared. Primary antibody (dilution 1:100) incubated sections were detected with biotinylated secondary antibody. The specimens were then exposed to DAB (3,3′-diaminobenzidine), counterstained with hematoxylin and mounted for confocal microscopy. The extent of scoring of the stained proteins was scaled between 0 and 3. Final scores were computed using a composite of intensity scores multiplied by the extent of staining score as described previously.^43^ The score of 1–4 was assessed as weak to moderate expression and 6–9 as strong expression.

### β-Galactosidase assay *in vitro*

Senescence-associated β-galactosidase (SA-β-Gal) assay was performed as per the standardized protocol.^44^ Briefly, the cells (5 × 10^4^) were plated and treated for 6 days. Post washing with phosphate-buffered saline, the cells were fixed with 4% paraformaldehyde. Accordingly, freshly prepared senescence-associated β-Gal staining solution (1 mg of 5-bromo-4-chloro-3-indolyl P3-D-galactoside (X-Gal) per ml in 40 mm of citric acid, sodium phosphate (pH 6.0), 5 mm potassium ferrocyanide, 5 mm potassium ferricyanide, 150 mm NaCl and 2 mm MgCl_2_) was used to stain the cells for 24–48 h. The stained cells were then fixed with methanol, air dried and observed under bright field microscope for senescent phenotype. Quantification was done with ImageJ software.

### Molecular docking

Molecular docking studies of 3-AWA with AKT were carried out using the Schrodinger suite 2015-1 molecular modeling software. The three-dimensional co-ordinates of AKT protein with Protein Data Bank ID: 3CQW^45^ having resolution 2 Å were downloaded from Protein Data Bank. The crystal structure of the protein is bound with the 5-(5-chloro-7H-pyrrolo[2,3-d]pyrimidin-4-yl)-4,5,6,7-tetrahydro-1H-imidazo[4,5-c]pyridine inhibitor with 42 nm activity. The co-ordinates of the bound inhibitor were used to define the active site of the protein for performing docking studies. To perform molecular docking studies, initially the protein was prepared and docking protocol was standardized.

### Molecular dynamic simulation

It is important to analyze the behavior of the protein–ligand complex in aqueous environment of the biological system. Therefore, molecular dynamics simulation studies, of the docked conformation with maximum affinity for the target protein, were done by Desmond software (version 4.1, Schrodinger, LLC, 2015).^46^ TIP3P solvent model with cubic boundary conditions of 10 Å distance was used, and the net charge of the system was neutralized. Production simulations were run in NPT ensemble with 1 bar pressure and 300 K temperature using Berendsen coupling scheme. Finally, 10 ns equilibration and production simulation were run for the protein–ligand complex and its co-ordinates were recorded after every 1.2 ps.

### *In vivo* study

Animal experiments were carried out according to the guidelines approved by the Institutional Animal Ethics Committee ‘CPCSEA’ (IAEC No. 51/02/15). To evaluate the *in vivo* antitumor activity of 3-AWA, seven healthy male Wistar rats (5–7 weeks old, b.w. 150–200 g) per group were taken. The animals were fed standard pelleted chow, sterile water and kept in a pathogen-free condition. The animals were randomized into three groups and six animals were taken per group. For the orthotopic induction of colorectal tumors, the animals were given an intrarectal instillation of 0.5 ml of freshly prepared 0.4% aqueous solution of *N*-methyl-N-nitrosourea (MNU) three times weekly for 3 weeks by a method as described previously.^47^ Twenty-two weeks after the initiation of administration of MNU, treatment regimens were started. The test group of animals received 10 mg/kg b.w. of 3-AWA, the positive control group received 25 mg/kg b.w. of 5-FU and the negative control group animals were given 0.9% normal saline solution in alternate days for another 3 weeks. The experiment was terminated at the end of 25 weeks and the animals were killed through anesthesia followed by cervical dislocation. The large bowel was dissected carefully throughout its length and observed for shape, size and the location of the tumors. The tumor volume was calculated from the equation: *V*=(*d*1 × *d*2)2 × 0.6, where *V* is the tumor volume, *d*1 is the largest diameter of the tumor and *d*2 is the smallest diameter of the tumor. None of the animals injected with tumor cells was excluded from the analysis. No statistical method was used to predetermine sample size. The experiments were executed and analyzed in a non-randomized and non-blinded manner.

### Statistical analysis

Graph Pad Prism version 5 (San Diego, CA, USA) was used to perform statistical analyses. Data were expressed as the mean±s.e. of three independent experiments. A two-sided value of *P*<0.05 was considered significant in all the cases. No statistical method was used to predetermine sample size. The experiments were executed and analyzed in a non-randomized and non-blinded manner. Comparisons between two groups were conducted using unpaired Student’s *t*-test. Experiments with more than two groups were compared by one-way analysis of variance followed by Tukey’s multiple comparison test. The tests were undertaken using SPSS, version 19.0, computer (Armonk, NY, USA).
