# Supplementary material for: AKT is indispensable for coordinating Par-4/JNK cross talk in p21 downmodulation during ER stress
Source: Oncogenesis. 2017 May 22;6(5):e341–. doi: 10.1038/oncsis.2017.41 (PMC5523074; doi:10.1038/oncsis.2017.41)
Supplement: Supplementary Information [file oncsis201741x1.pdf]

AKT is indispensable for coordinating Par-4/JNK cross-talk in p21 downmodulation during ER stress

Reyaz ur Rasool,[1,2] Debasis Nayak,[1,2] Souneek Chakraborty,[1,2] Mir Mohd Faheem,[2] Bilal Rah,[3] Priya Mahajan,[4] Veena Gopinath,[5] Archana Katoch,[1,2] Zainab Iqra,[2] Syed Khalid Yousf,[6] Debaraj Mukherjee,[6] Lekha Dinesh Kumar,[5] Amit Nargotra,[4] Anindya Goswami[1,2,*]

[1] Academy of Scientific & Innovative Research (AcSIR), CSIR- Indian Institute of Integrative Medicine, Jammu 180001, India

[2] Cancer Pharmacology Division, CSIR-Indian Institute of Integrative Medicine, Jammu 180001, India

[3] University of Nebraska Medical Center (UNMC), Omaha, NE 68198, USA

[4] Discovery Informatics Division, CSIR- Indian Institute of Integrative Medicine, Jammu 180001, India

[5] Cancer Biology, CSIR-Centre for Cellular & Molecular Biology, Hyderabad 500007, India

[6] Natural Product Chemistry Division, CSIR- Indian Institute of Integrative Medicine, Jammu 180001, India

[*]Corresponding Author:

Anindya Goswami, PhD

CSIR-Indian Institute of Integrative Medicine,

Canal Road, Jammu 180001, India

Tel.: +91 0191 2569111; fax: +91 0191 2569333
E-mail: agoswami@iiim.ac.in

Supporting Information Figure 1.

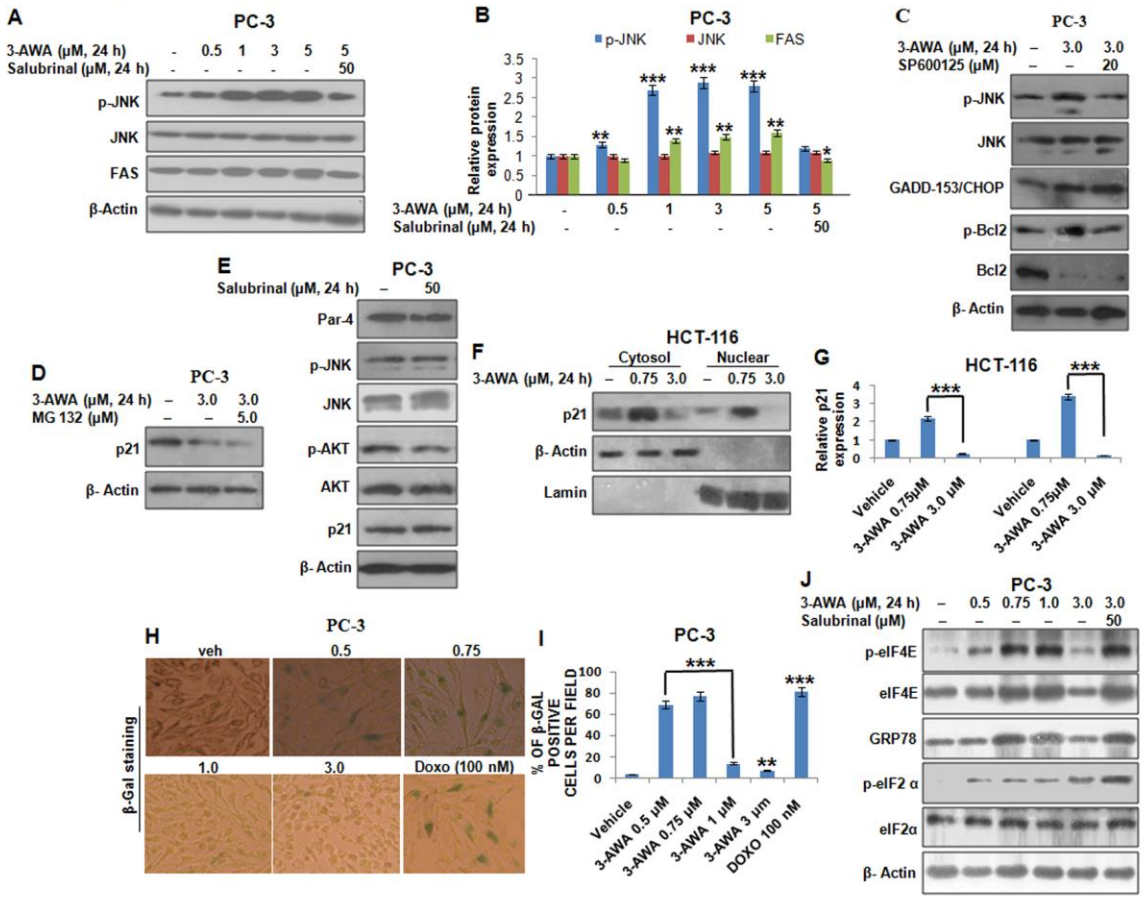

Supporting Information Figure 1. JNK activation and p21$^{Cip1/WAF1}$ down-regulation by 3-AWA. (A, B) PC-3 cells were treated with increasing concentration of 3-AWA, as indicated, in presence or absence of Salubrinal. The lysates prepared were analyzed through western blotting for the expression of p-JNK, JNK and FAS. (C) PC-3 cells were exposed to 3 µM 3-AWA and/or Salubrinal and analyzed for the expression of the indicated proteins. (D) The cells were treated with 3-AWA and/or a proteasomal inhibitor MG 132 and the expression of p21 was analyzed through western blotting. (E) Effect of Salubrinal was studied on the expression of the indicated proteins. (F, G) Fractionation of cytosolic and nuclear proteins was carried-out in HCT-116 p53$^{+/+}$ cells. The fractions obtained were analyzed through western blotting, as shown, for p21 expression. Actin and lamin were used as cytosolic and nuclear control. The data shown are the representative of three independent experiments. (H, I) PC-3 cells were

exposed to the indicated concentrations of 3-AWA or Doxorubicin (positive control), β-Gal staining analyses was done as described in material methods. (J) PC-3 cells were treated with the indicated concentrations of 3-AWA and/or Salubrinal and the expression of the given proteins was studied with their specific antibodies. In all the western blotting experiments β-Actin was used as loading control. For data analysis One-way ANOVA was used, bar graphs are mean ± s.e.m. The data shown are the representative of three independent experiments. ****P*≤0.001; ***P*≤0.01; **P*≤0.05.

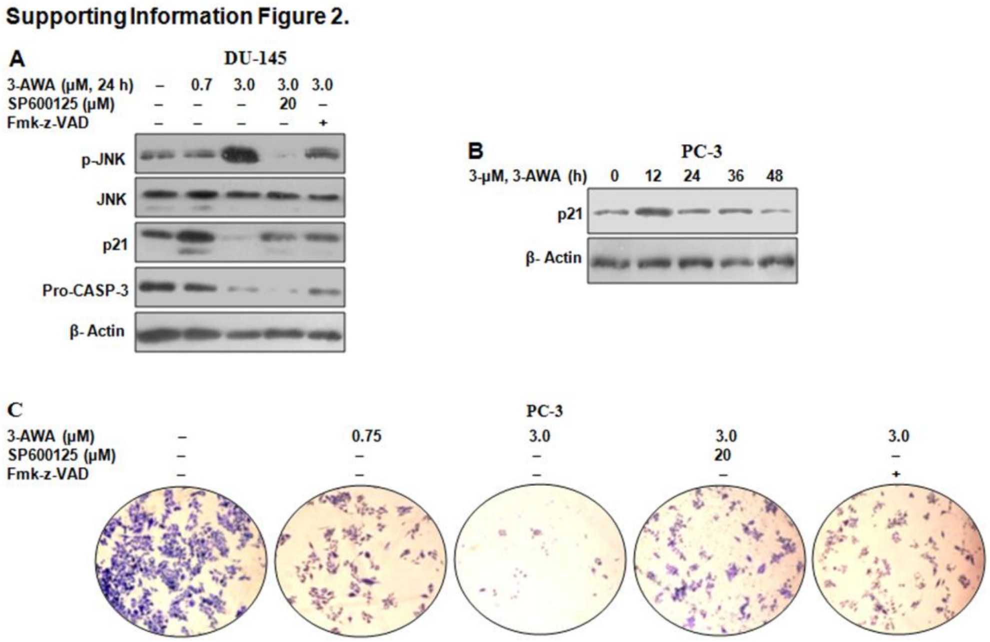

Supporting Information Figure 2. 3-AWA decreases p21 expression through JNK/caspase-3/p21 axis and inhibits colony formation. (A) Lysates prepared from DU-145 cells, treated with given concentrations of 3-AWA and/or SP600125 or Fm-z-VAD for the indicated time point, were analyzed through immunoblotting for the expression pattern of p-JNK, JNK, p21 and caspase-3. (B) Time-point dependent analysis of 3-AWA was carried out on the expression of p21 through western blotting. β-Actin was used as loading control. (C) Colony formation assay was carried out in PC-3 cells following treatment with 3-AWA and/or SP600125 or Fm-z-VAD as indicated.

Supporting Information Figure 3.

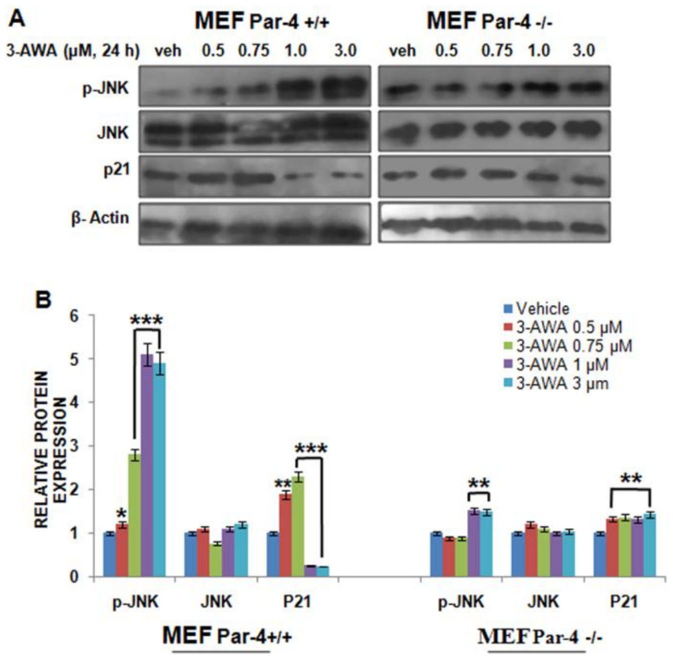

Supporting Information Figure 3. Down-regulation of p21 by 3-AWA is Par-4 dependent. (A) MEF Par-4$^{+/+}$ and MEF Par-4$^{-/-}$ cells following treatment with the increasing concentration of 3-AWA for the indicated time points, were examined for the expression of p-JNK, JNK and p21 through western blotting. β-Actin was used as loading control. Students's t-test and One-way ANOVA was used for statistical significance; bar graphs are mean ± s.e.m. The data shown are the representative of three independent experiments. ***P≤0.001; **P≤0.01; **P≤0.05.

Supporting Information Figure 4.

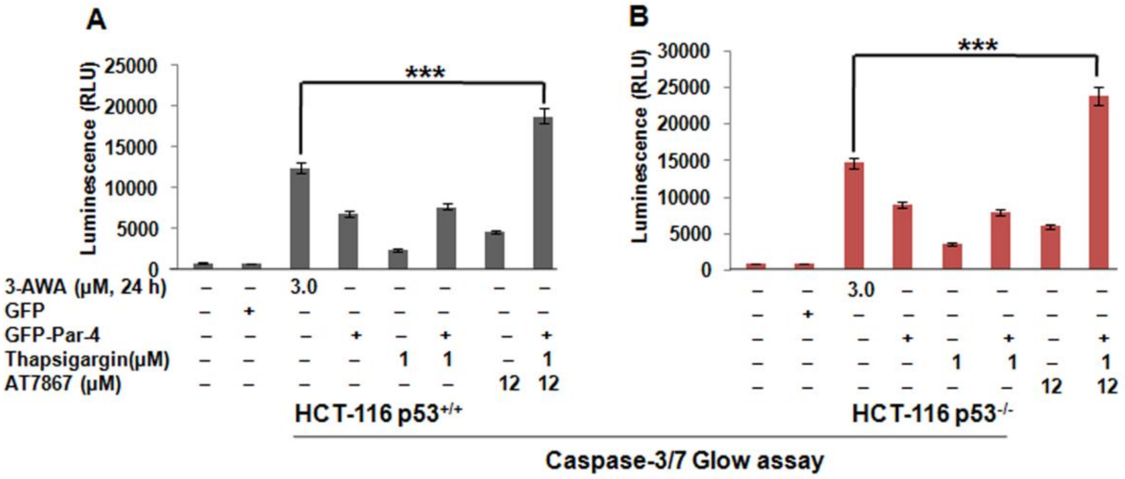

Supporting Information Figure 4. p21 downregulation by 3-AWA is associated with increased caspase-3/7 activation. (A, B) HCT-116 p53$^{+/+}$ and HCT-116 p53$^{-/-}$ cells were transfected with GFP/GFP-Par-4 and/or treated with the indicated concentrations of 3-AWA, AT7867 and Thapsigargin in combination or alone to check the activation of caspase-3/7 through Caspase Glow kit. Student's t-test and One-way ANOVA with SAS 9.2 were used for data analysis, bar graphs are mean ± s.e.m. Data are the mean of three independent experiments. ***$P \leq 0.001$.

Supporting Information Table

Table: Body weight monitoring of animals

	Body weight in grams				
**Treatment groups**	**At wk 5**	**At wk 10**	**At wk 15**	**At wk 20**	**At wk 25**
Normal saline (NS)	175 ± 3.5	189 ± 2.1	207 ± 2.6	228 ± 3.3	242 ± 4.6
5-FU (25 mg/kg, b.w.)	172 ± 2.4	184 ± 2.9	201 ± 2.1	216 ± 3.2	223 ± 2.5
3-AWA (10 mg/kg, b.w.)	170 ± 1.8	181 ± 3.2	203 ± 2.7	219 ± 4.1	229 ± 3.7

Body weight of rats are represented as average ± sem

Supporting Information Table. Body weight monitoring of animals in grams. The Body weight of rats are represented as average ± sem.
